# Gene design, fusion technology and TEV cleavage conditions influence the purification of oxidized disulphide-rich venom peptides in *Escherichia coli*

**DOI:** 10.1186/s12934-016-0618-0

**Published:** 2017-01-17

**Authors:** Ana Filipa Sequeira, Jeremy Turchetto, Natalie J. Saez, Fanny Peysson, Laurie Ramond, Yoan Duhoo, Marilyne Blémont, Vânia O. Fernandes, Luís T. Gama, Luís M. A. Ferreira, Catarina I. P. I. Guerreiro, Nicolas Gilles, Hervé Darbon, Carlos M. G. A. Fontes, Renaud Vincentelli

**Affiliations:** 1 CIISA-Faculdade de Medicina Veterinária, Universidade de Lisboa, Avenida da Universidade Técnica, 1300-477 Lisbon, Portugal; 2 NZYtech Genes & Enzymes, Campus do Lumiar, Estrada do paço do Lumiar, 1649-038 Lisbon, Portugal; 3 Unité Mixte de Recherche (UMR) 7257, Centre National de la Recherche Scientifique (CNRS)–Aix-Marseille Université, Architecture et Fonction des Macromolécules Biologiques (AFMB), Marseille, France; 4 Institute for Molecular Bioscience, The University of Queensland, St Lucia, 4072 Australia; 5 CEA/DRF/iBiTecS, Service d’Ingénierie Moléculaire des Protéines, 91191 Gif-Sur-Yvette, France

**Keywords:** Venom peptides, Gene design, Recombinant expression, Periplasm, Disulphide-rich peptides, Fusion protein, *Escherichia coli (E. coli)*, High-throughput expression

## Abstract

**Background:**

Animal venoms are large, complex libraries of bioactive, disulphide-rich peptides. These peptides, and their novel biological activities, are of increasing pharmacological and therapeutic importance. However, recombinant expression of venom peptides in *Escherichia coli* remains difficult due to the significant number of cysteine residues requiring effective post-translational processing. There is also an urgent need to develop high-throughput recombinant protocols applicable to the production of reticulated peptides to enable efficient screening of their drug potential. Here, a comprehensive study was developed to investigate how synthetic gene design, choice of fusion tag, compartment of expression, tag removal conditions and protease recognition site affect levels of solubility of oxidized venom peptides produced in *E. coli*.

**Results:**

The data revealed that expression of venom peptides imposes significant pressure on cysteine codon selection. DsbC was the best fusion tag for venom peptide expression, in particular when the fusion was directed to the bacterial periplasm. While the redox activity of DsbC was not essential to maximize expression of recombinant fusion proteins, redox activity did lead to higher levels of correctly folded target peptides. With the exception of proline, the canonical TEV protease recognition site tolerated all other residues at its C-terminus, confirming that no non-native residues, which might affect activity, need to be incorporated at the N-terminus of recombinant peptides for tag removal.

**Conclusions:**

This study reveals that *E. coli* is a convenient heterologous host for the expression of soluble and functional venom peptides. Using the optimal construct design, a large and diverse range of animal venom peptides were produced in the µM scale. These results open up new possibilities for the high-throughput production of recombinant disulphide-rich peptides in *E. coli*.

**Electronic supplementary material:**

The online version of this article (doi:10.1186/s12934-016-0618-0) contains supplementary material, which is available to authorized users.

## Background

Animal venoms comprise an arsenal of dozens to hundreds of structurally diverse disulphide-rich peptides that possess important pharmacological, therapeutic and biotechnological value. Considering the number of animal species that produce venoms and the average number of peptides per venom, the library of naturally evolved venom peptides may encompass millions of different molecules. These highly stable disulphide-reticulated peptides display formidable affinity and selectivity while presenting low immunogenicity making them attractive candidates for the development of novel therapeutics [1]. In general venom peptides target a variety of cell surface receptors, such as ion channels, and interaction with their molecular ligands dramatically affects cellular function [2]. Venom peptides generally contain between 20 and 120 residues and include up to eight disulphide bonds that are critical for both biological activity and stability. Thus, the correct oxidation of cysteine residues leading to proper disulphide pairing is required for folding and functional activity. Unfortunately, the use of venom peptides as therapeutic or biotechnological molecules is still hampered by the difficulty to produce native and active proteins in sufficient amounts [3].

De novo gene synthesis is the most convenient route to obtain genes for recombinant expression. This is particularly true for genes encoding venom peptides, as the sequence information from genomic and transcriptomic projects is usually not available as palpable DNA. Designing a gene to express a protein requires selecting from an enormous number of possible DNA sequences [4]. In addition, effective gene design may be affected by gene family. For example, a high percentage of cysteine residues in venom peptides may impose particular constraints in levels of gene expression and these remain to be uncovered for the particular case of genes encoding animal venom peptides. Usually, gene design involves selecting a codon usage that maximizes levels of expression based on the codon bias of a subset of highly-expressed native host genes [5, 6]. Expression may also be impaired by a strong mRNA secondary structure near the translational start site, inadequate GC content or presence of unwanted regulatory sequences recognized by the cellular expression machinery [7]. Although different studies have analysed how genes can be designed efficiently, there is still no information about the major factors affecting expression of genes encoding reticulated peptides in heterologous hosts.

*Escherichia coli* is a highly robust bioreactor for heterologous protein expression. Several high throughput platforms have been developed using this bacterium [8, 9]. *E. coli* is particularly adequate to generate large libraries of recombinant proteins to apply to functional screens with biomedical and biotechnological relevance. However, production of disulphide-bonded proteins in bacteria is hampered by the lack of an effective post-translational system. Thus, in *E. coli* reticulated peptides are especially prone to aggregation or degradation due to possible mispairing of cysteine residues or undesirable intermolecular disulphide bonds. In addition, gene expression in bacteria is regulated by strong promoters, leading to the accumulation of recombinant proteins as insoluble aggregates or inclusion bodies. Different technologies have been developed to promote the correct oxidation of cysteine residues in recombinant proteins expressed in bacteria [10]. Exporting the proteins to the *E. coli* oxidative periplasm is a well-established strategy although levels of recombinant protein can be limited by protein export [11]. For successful expression, two challenges must be met; (i) the peptide of interest must be maintained in a soluble state, and (ii) the correct disulphide bonds must be formed within the peptide. Recently some fusion tags displaying not only a solubilizing effect but also redox properties, such as DsbA and DsbC, were described by our group to enhance the solubility of venom peptides while promoting correct disulphide bond formation [8, 11]. However, the most effective high-throughput-compatible strategy to express a wide panel of correctly folded venom peptides in *E. coli* remains to be established.

Fusion tags are indispensable tools for protein expression and purification in bacteria [12]. However, presence of a fusion tag may interfere with protein function and their removal from the target protein is desirable. Tobacco etch virus (TEV) protease [13] is one of the most popular enzymes used to remove fusion tags from recombinant proteins due to the stringent sequence specificity it displays. However, TEV protease may require a Gly or Ser residue at the C-terminus (P1′ position) of its recognition site [14], leaving a non-native Ser or Gly residue at the N-terminus of the target protein after tag removal. In the specific case of venom peptides it is well known that the N-terminal part of the peptide can contribute to the pharmacophore involved in receptor binding and thus the presence of an N-terminal fusion tag may affect biological activity [15]. Thus, removal of N-terminal tags is absolutely required to guarantee functional recombinant venom peptides.

The European VENOMICS Project (FP7, n° 278346) is a consortium studying animal venoms to develop the use of toxins as innovative drugs. VENOMICS aims to establish a new paradigm in venom science by first exploring the peptide content of venoms by transcriptomics and proteomics and then by building a large library of bioactive molecules for drug discovery by high-throughput synthesis and recombinant expression of animal venom peptides. For the recombinant production of oxidized peptides in *E. coli*; first, the consortium reproduced and benchmarked several production options published in the literature and then, after optimization, applied the best protocol to the production of 5000 recombinant animal venom peptides selected from the VENOMICS database. The optimization of the production protocol is detailed in this manuscript. The application of this new procedure for the production of 5000 toxins is described in the accompanying article.

Within this publication, we have examined and optimized gene design, the choice of fusion tag, as well as TEV cleavage conditions and recognition site to improve the production of oxidized recombinant venom peptides in *E. coli*. The challenge to face was to find the best protocol that would be amenable to the throughput dictated by the VENOMICS objectives; build a peptide library of thousands of reticulated animal venom peptides in a matter of months, where individual optimization of the production steps would be impossible.

Overall data reported here suggest that *E. coli* is an effective host to express milligram per litre culture quantities of correctly oxidized recombinant venom peptides using high-throughput technologies.

## Methods

### Design of gene variants encoding venom peptides

For the initial studies, 24 representative venom peptides originating from 21 different animal species were selected. The peptides had sizes ranging from 21 to 84 residues and contained between 2 and 7 disulphide bridges (Additional file 1: Table S1). The primary sequence of the 24 venom peptides was back-translated using a Monte Carlo repeated random sampling algorithm to generate three gene variants per peptide. This algorithm selects a codon for each position at a probability defined in a codon frequency lookup table. The lookup table applied to create the three variant designs of each gene varied in global codon usage within codons used preferentially in highly expressed or average native *Escherichia coli* genes. Other factors considered for gene design were GC content, mRNA structure, absence of prokaryotic regulatory sequences and contiguous strings of more than 5 identical nucleotides, which were set not to vary within the different gene variants. Due to the use of Monte-Carlo sampling for gene design, all the three variants were significantly different in sequence identity from each other. The average pairwise DNA sequence identity was 79.8% for the 24 datasets. Thus, in the initial phase of this work a total of 72 genes were designed (3 gene variants of 24 peptides), the sequences presented in Additional file 1: Table S1.

### Gene synthesis, cloning and protein expression/purification of initial 72 variants

The 72 synthetic gene variants were produced using standard procedures [16, 17]. The sequence coding for a TEV protease cleavage site (ENLYFQ/G) was engineered upstream of each gene. This sequence was identical for all 72 gene variants. Nucleic acids were synthesised containing Gateway recombination sites on each extremity. After PCR assembly, synthetic genes were directly cloned into pDONR201 using Gateway™ BP cloning technology (Invitrogen, USA) [18]. Like for all the other plasmids and constructs used in this study, each construct was completely sequenced in both directions to ensure 100% consistency with the designed sequences. The 72 sequence entry clones were recombined using the Gateway™ LR cloning technology (Invitrogen, USA) to transfer the peptide-coding genes into pETG82A destination vector [19]. Destination vector pETG82A contains the sequence coding for a DsbC fusion partner, which is located at the 5′end of the inserted gene. All recombinant peptides fused with an N-terminal DsbC fusion tag contain an additional internal 6HIS tag for protein purification. Each variant plasmid was used to transform *E. coli* expression host strain BL21(DE3) pLys S (Invitrogen, USA). The choice of the plasmid and strain used in this experiment was based on our previous studies done on reticulated peptides [11]. Transformed cells were grown on solid media and resulting colonies were used to inoculate 4 mL of ZYP-5052 auto-induction medium [20] supplemented with 200 µg/mL of ampicillin. All steps were carried out in 24 deep-well plates (DW24) following exactly the lab standard protocol [11, 21], which is described briefly below. ZYP-5052 medium is an auto-inducing buffered complex medium. Recombinant protein expression was induced following a standardized two-step process. Cells were grown at 37 °C to quickly reach the glucose depletion phase just before the induction. After that step (4 h, OD_600_ ~1.5) the temperature was lowered to 17 °C for 18 h to favour protein folding and soluble protein expression. Cells were collected by centrifugation, re-suspended in 1 mL of lysis buffer (Tris 50 mM, NaCl 300 mM, Imidazole 10 mM, Lysozyme 0.25 mg/mL, pH 8) and recombinant proteins purified from crude lysates using an automated nickel affinity procedure [8, 9]. Briefly, the crude cell lysates were incubated with Sepharose chelating beads (200 μL with bound Ni^2+^) and then transferred into 96-well filter plates (Macherey-Nagel). The wells were washed twice with buffer A (Tris 50 mM, NaCl 300 mM, Imidazole 50 mM, pH 8). The recombinant fusion proteins were eluted from the resin beads with 500 µL of elution buffer (Tris 50 mM, NaCl 300 mM, Imidazole 250 mM, pH 8) into 96-deep-well plates. All protein purification steps were automated on a Tecan robot (Switzerland) containing a vacuum manifold. Analysis of the purified protein yields was performed on a Labchip GXII (Perkin Elmer, USA) microfluidic high throughput electrophoresis system. These analyses provided an estimation of the molecular weight, purity and concentration of the proteins. All the quantitative values given in this manuscript are based on the calculation made by the Labchip GXII software.

### Construction of pHTP-derivative vectors to express venom peptides in *E. coli*

A collection of 5 novel vectors was constructed based on the prokaryotic expression vector pHTP1 (NZYTech, Portugal). The DNA sequences encoding a fusion protein tag were inserted into pHTP1 plasmid downstream of the T7 promoter, such that the protein tags would become fused to the N-terminus of the target peptide. DNA sequences encoding fusion tags were obtained by gene synthesis (see above) and included upstream and downstream NcoI restriction sites. Once inserted into pHTP1 backbone after digestion with NcoI, the five pHTP vectors retained the C-terminal hexa-histidine (6HIS) tags for protein purification (Additional file 2: Table S2). The five novel tags were based on disulphide-bond isomerase C (DsbC) and maltose-binding protein (MBP) [11, 21–28] sequences, some of the best tags for producing functional venom peptides in *E. coli* described to date. Thus, vector pHTP2 (pHTP-LLDsbC) encodes the sequence of DsbC for cytoplasmic expression. In addition, pHTP3 (pHTP-mutDsbC) express a redox inactive mutant of DsbC while in pHTP4 (pHTP-DsbC), the sequence of a signal peptide is included before the DsbC to allow export of the recombinant fusion protein to the periplasm. Similar vectors were also produced encoding MBP derivatives and were termed pHTP5 (pHTP-LLMBP) and pHTP6 (pHTP-MBP), respectively. The protein sequences of the six fusions created for this project are presented in Additional file 2: Table S2. Schematic representations of the fusion proteins expressed from each vector are shown in Fig. 3.

### Cloning genes encoding 16 venom peptides into 6 pHTP vectors

The genes encoding 16 representative animal venom peptides were synthesised as described previously with a codon usage optimized for expression in *E. coli*. Seven selected peptides are the same as those selected in Additional file 1: Table S1 (Additional file 3: Table S3, in bold). Out of these 16 peptides, 8 were selected for the TEV cleavage optimization protocol (Additional file 3: Table S3, in italic) including MT7, that was also selected in this study as it turned out to be one of the most challenging target of our previous study [11]. The 16 synthetic genes encoding venom peptides were directly cloned into pUC57. Upstream and downstream of all 16 genes, a 16 bp sequence was engineered to allow cloning into vectors of the pHTP-series using the NZYEasy cloning protocol (NZYTech, Portugal). Sequence and properties of the 16 genes produced here are presented in Additional file 3: Table S3. The 16 different peptide genes were transferred from the pUC57 vector into each one of the 6 expression vectors in an experiment consisting of 96 cloning reactions. Reactions consisted of 240 ng of each linearized vector, 120 ng of the pUC57 derivative containing the target peptide gene, 1 μL of enzyme mix and 2 μL of 10× reaction buffer. Cloning reactions were carried out in 20 μL final volume on a thermal cycler programmed as follows: 37 °C for 1 h; 80 °C for 10 min and 30 °C for 10 min. The reaction mixtures were used to transform DH5α *E. coli* competent cells. Two colonies were picked for each construct and the presence of insert confirmed by PCR using the vector specific T7 and pET24a forward and reverse primers, respectively. All 96 plasmids containing the venom peptide genes were sequenced to confirm integrity of the cloned nucleic acid.

### Recombinant protein purification and TEV cleavage protocol

The 96 recombinant pHTP derivatives were used to transform BL21 (DE3) pLysS *E. coli* cells. Recombinant strains were grown in 4 mL of auto-induction medium supplemented with kanamycin (50 μg/mL). Recombinant peptides fused with different tags were purified as described above.

The TEV clone used in these studies is the TEV_SH_, a kind gift of Dr H. Berglund [29]. The purification of the TEV_SH_ was done following the published protocol except that the LB medium was replaced by ZYP5052 (or TB) medium to reach a yield of purified TEV_SH_ up to 100 mg/L culture. At the end of the purification, the TEV_SH_ was dialyzed into Hepes 20 mM, NaCl 300 mM, Glycerol 10% (v/v), pH 7.4 to remove traces of DTT, concentrated to 2 mg/mL and stored at −80 °C.

The TEV cleavage protocol used here to remove fusion tags from recombinant peptides was described elsewhere [9]. In order to simplify the study, based on previous in-house experiments [8, 9, 11], several parameters were kept constant (unless this is specified) for all the TEV cleavages; the concentration of purified fusion protein (1 mg/mL), a fusion/TEV ratio of 1/10 (w/w), the buffer composition, the temperature (30 °C) and the incubation period (18 h). The cleavage buffer chosen was the IMAC protein elution buffer (Tris 50 mM, NaCl 300 mM, Imidazole 250 mM, pH 8) which considerably simplifies downstream processing by avoiding the dialysis step to remove the imidazole from the buffer prior to the addition of the protease. When necessary, the cleavage buffer was supplemented with fresh DTT (see “Results” section).

Before the cleavage an aliquots (20 µL) of the 96 un-cleaved samples were blocked with caliper sample buffer following the manufacturer protocol. After the 18 h TEV cleavage, samples were acidified for 1 h with 5% ACN, 0.1% formic acid. Precipitated material (TEV protease, fusion tags and misfolded peptides) was removed by centrifugation (10 min at 4100×*g*). Three Aliquots (20 µL) of the 96 cleaved samples were collected, two for the mass spectrometry analysis and one that was boiled with the caliper sample buffer. To check that the cleavage was successful and to calculate TEV cleavage yields, these samples were run side by side with the uncleaved controls on the caliper GXII system. The TEV cleavage efficiency was calculated using the Labchip quantification software. Because proteins below 5 kDa cannot be quantified by the software, the cleavage efficiency was only calculated by integrating and comparing the disappearance of the fusion-peptide species band on the labchip.

### Tag removal and mass spectrometry

After TEV cleavage, one aliquot (20 µL) of the 96 cleaved samples kept for mass spectrometry were analysed in-house on a reverse phase C18 column at 37 °C (Hypersil GOLD 50 × 1.0 mm, 1.9 μm, 175 Å, ThermoScientific) at a flow rate of 200 μL/min on a UHPLC–MS with electrospray detection (Accela High Speed LC system with detector MSQ+ , ThermoScientific, San Jose, CA). The gradient slope (solvent A: water, B; acetonitrile, both solvents containing 0.1% formic acid) went from 5 to 40% B in 2 min followed by an 80% wash and re-equilibration (total time: 6 min). MS acquisition was performed in the positive ion mode from *m/z* 100 to 2000. To confirm correct peptide molecular weight, the resulting mass spectra were de-convoluted using manual calculations. The isotopic pattern measured was compared with the theoretical one determined from the amino acid sequences using DataExplorer software (Version 4.9, Applied Biosystems). The quantitative calculation of peptide yields were determined using automatic processing with Xcalibur software (ThermoScientific), by OD_280 nm_ measurement and peak areas integration. The peptide cleavage and recovery yield calculation was made by comparing the quantities of peptide present in the initial sample after Nickel purification (fusion-His-Toxin band on the caliper), given by the caliper GX II software versus the final yield of oxidized peptide quantified by the OD_280 nm_ measurement and peak areas integration by the LC system. To comfort these results, the second aliquot was sent to our VENOMICS collaborator Dr. L. Quinton (University of Liège, Belgium) toxin and mass spectrometry specialist who confirmed the correct oxydation and good cystein connectivity of these known toxins (data no shown). More details on the quality control by mass spectrometry on the VENOMICS toxins can be found on the accompanying article.

### Generation of N-terminal variants of DNA/RNA-binding protein KIN17

To test the efficacy of TEV protease to cleave peptide chains including variations at the C-terminus of the consensus recognition site of the enzyme (ENLYFQ/X), the gene encoding the C-terminal domain of the DNA/RNA-binding protein Kin17 (Kin17´) from *Homo sapiens* was synthetized. PCR was used to create 20 gene variants encoding derivatives of the Kin17´ protein with 20 different N-terminal amino acids at the TEV recognition site. The genes were produced by PCR including the reverse primer HSr, 5′-GGGGACCACTTTGTACAAGAAAGCTGGGTCTTATTAAAGTTTAGAGATGTCTTCAT-3′ and the forward primers presented in Additional file 4: Table S4. Amplified nucleic acids contained Gateway recombination sites on each extremity. Thus, genes were initially directly cloned into pDONR201 using Gateway™ BP cloning technology (Invitrogen, USA). The 20 gene variants were subsequently cloned into pDest17 (Invitrogen, USA) using Gateway™ LR cloning technology (Invitrogen, USA). Resulting expression plasmids encode for Kin17′ derivatives containing an N-terminal 6HIS tag and a TEV recognition site combining 20 variations at the residue occupying its C-terminal (P1′) position. Primary sequence of both proteins and respective genes are presented in Table S4. The 20 plasmid derivatives were used to transform BL21 (DE3) pLysS *E. coli* cells and recombinant proteins were produced, purified and cleaved. The TEV cleavage of the 20 variants of Kin17 was done in the buffer and conditions selected for the final venom peptide production pipeline of the FP7 VENOMICS Project.

## Results

### Codon usage of venom peptide-coding genes cause expression differences

Twenty-four genes of various lengths encoding venom peptides from different species and containing different numbers of disulphide bridges were chosen to explore the effects of codon usage on soluble levels of purified proteins. These genes encode venom peptides that are evolutionarily, structurally and functionally diverse. The experiment aims to evaluate if subtle changes in codon usage can affect levels of recombinant peptide expression in *E. coli*. Three variants of each gene were initially designed by back-translating venom peptide sequences using a Monte Carlo repeated random sampling algorithm to select codons probabilistically from codon frequency lookup tables. The codon usage of the 72 devised genes (3 variants of 24 genes) is presented in Additional file 5: Table S5 and reflects the codon usage of *Escherichia coli* genes expressed at moderate to high levels. However, created gene variants incorporated changes in DNA primary sequences which reflect the random sampling of codon selection and the overall freedom permitted by the algorithm used for gene design. Thus, the average pairwise DNA sequence identity was 79.8% within the three variants of the 24 datasets.

**Fig. 1:**
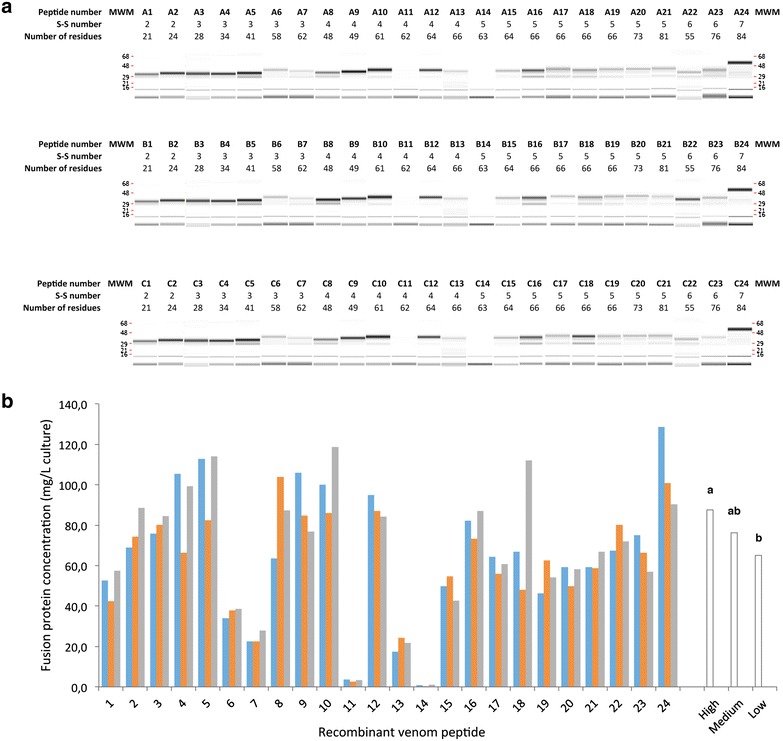
Yields of 24 purified recombinant fusion proteins originated from 3 different gene designs. **a** Virtual gel showing the expression levels of 24 recombinant peptides obtained from gene design A, B and C that were purified through IMAC and evaluated using the Labchip GXII (Caliper, USA). **b** Comparison of expression levels of variant A (*blue*), variant B (*orange*) and variant C (*grey*) of the 24 recombinant peptides. On the* right* are representations of the means for high, medium and low expressing variants calculated for the 16 fusion peptides produced at higher yields. Means without a common letter differ at *P* < 0.01

The 72 genes were synthesized and cloned using the Gateway system into pETG82A prokaryotic expression vector under the control of a T7 promoter and in fusion with the gene encoding the DsbC fusion tag for cytoplasmic expression. *E. coli* BL21 (DE3) pLys S were transformed with the 72 plasmids and grown in auto-induction media. Fusion proteins were purified and protein integrity and yield measured by Caliper Labchip GXII analysis (Fig. 1a). Depending on the peptide of interest, the purified fractions run on the Caliper Labchip GXII mainly as a single band (peptides 1, 2, 3, 4…) or as a double band (5, 8, 16, 18…). The single band represents the good protein population (His-DsbC-peptide) while the lower band (around 29 kDa) corresponds to the His-DsbC protein alone after truncation/degradation of the target peptide. This lower band probably indicates that there is a portion of the peptide population that was not properly folded and was degraded during the expression or purification processes. The protein concentration depicted in Fig. 1b has been calculated by integrating only the His-DsbC-peptide band using the Caliper software. The data, presented in Fig. 1b, revealed that yields of purified fusion protein varied from ∼1 mg/L (for fusion protein 14) to above 100 mg/L (for fusion proteins 4, 5, 8, 9, 10, 18 and 24). For the vast majority of targets (19/24) the quantities of fusion protein purified would allow the purification of milligram scale of target peptide per litre of culture (assuming a cleavage and purification yield around 100%) while for the remaining five peptides (6, 7, 11, 13 and 14) a larger volume of culture would be needed.

The correlation between primary sequence of gene variants and properties that have been suggested to affect expression was analysed. Deleterious motifs, such as 5′ mRNA secondary structures could not have affected levels of expression as venom genes were all fused to the same 5′-prime sequence, which encodes the protein fusion tag. There was no correlation between protein expression and number of disulphide bridges, peptide size, CAI value and GC content (data not shown). This suggests that differences in gene expression were determined by other sequence related properties in particular by codon usage. In order to investigate how changes in codon usage affected levels of recombinant peptides, the relation between protein yields of the low, medium and high expresser variants within the 24 data sets were compared. Fusion proteins expressing at lower levels, which concern peptides 6, 7, 11, 13 and 14 (Fig. 1), were excluded from the analysis. The data revealed that protein yields of the high, medium and lower expresser variants of the peptides analysed were significantly different. Thus, lower expressers produced on average 65.1 mg/L of recombinant fusion protein, while fusion protein yields of higher expressers were, on average, of 87.55 mg/L (Fig. 1b). These differences are significantly different (*p* = 0.01).

**Fig. 2:**
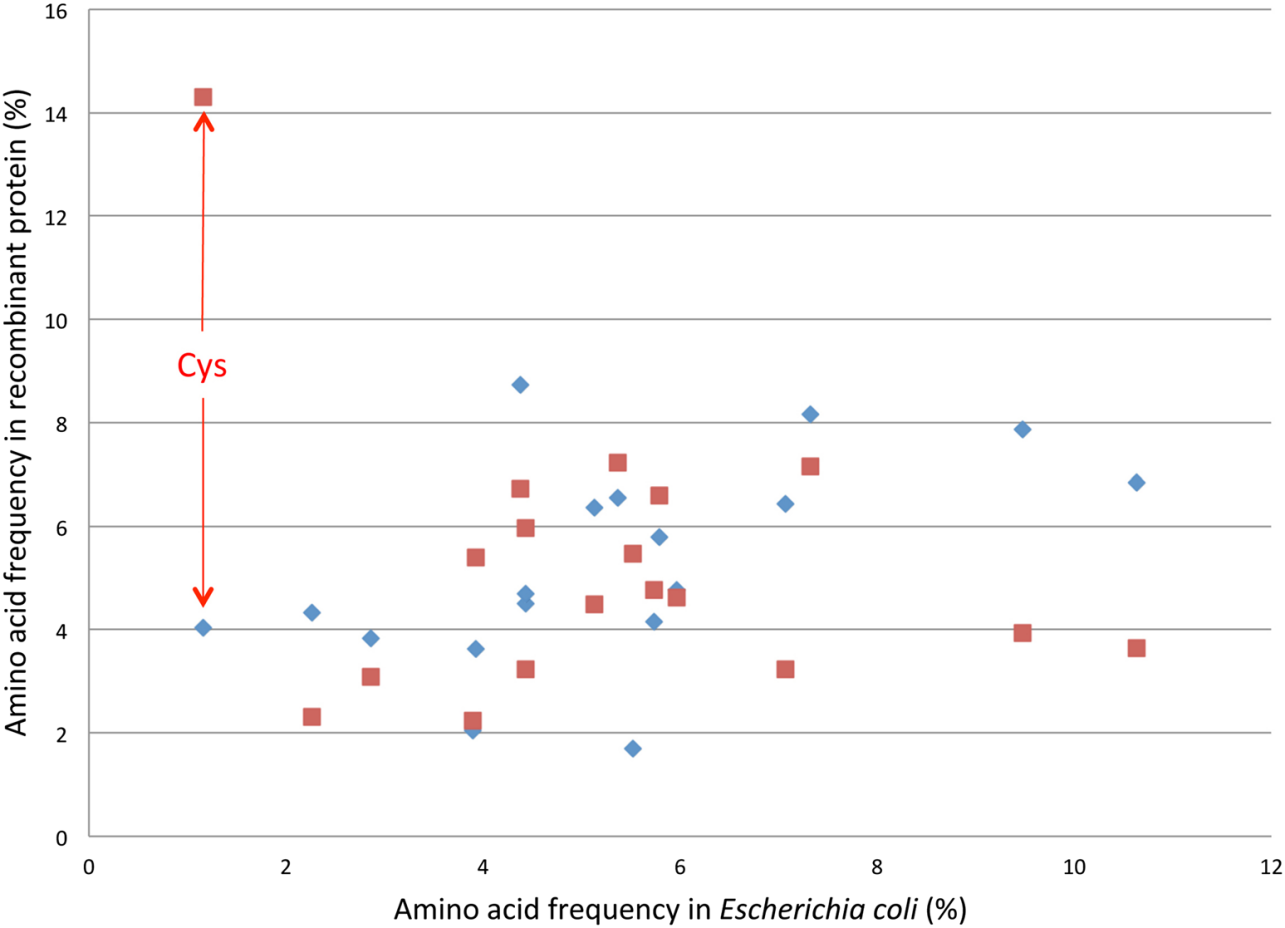
Comparison of amino acid frequency in *Escherichia coli* with the frequency of each amino acid in recombinant peptides analysed in this study. Percentage of abundance of each amino acid in *E. coli* is displayed in *blue*. In *red*, frequency of the same amino acid in venom peptide coding genes analysed in this study excluding the sequence encoding the fusion tag. A dramatic increase in the percentage of the amino acid cysteine is observed in venom peptides and this is highlighted by a *red arrow*

To evaluate what differences in codon usage could explain observed differences in protein expression, the codon usage of low and high expressing variants was compared. Codon usage tables including genes containing the fusion tag are presented in Table S6. Major differences in codon usage concern in particular one amino acid, cysteine, although slight changes were also observed for other residues in particular arginine, asparagine, glutamate, histidine, isoleucine, phenylalanine and serine. Summary codon usage data for these 8 amino acids is shown in Table 1. The codon bias observed for low expressing genes revealed a preference for Cys-TGC codon while in high expressing genes Cys-TGT is favoured. In addition, in low expressing genes Cys-TGC is used 1.38 times more frequently than Cys-TGT, while in high expressing genes Cys-TGT is only used 1.04 times more often than Cys-TGC. Although other factors may eventually be operating, this observation suggests that high expression of genes encoding peptides requires a similar contribution of both Cys-TGC and Cys-TGT codons, suggesting that a higher percentage of one codon compared to the other will affect expression. Cysteine codon usage in *E. coli* also points to a more balanced utilization of the two codons (Table 1). To investigate factors that may explain this observation, amino acid frequency in *E. coli* genes and within the 24 venom peptides selected for this study and their associated fusion proteins were compared. The data, presented in Fig. 2, revealed that cysteine is ~12.5 and 3.5 times more frequent in venom peptides (14.3%) and in the recombinant fusion proteins (4.1%), respectively, than in *E. coli* (1.16%). Thus, the data suggest that expression of venom peptides at high levels is favoured by the presence of the two cysteine codons at similar frequency in synthetic genes. This should avoid the depletion of one codon when genes are expressed at very high levels.

**Table 1:** Codon usage of genes encoding high and low expresser variants encoding either venom peptides or the respective fusion protein

AA	Codon	*Fc*, HE, toxins	*Fc*, LE, toxins	*Fc*, HE, fusion	*Fc*, LE, fusion	*Fc, E. coli*
Arg	AGA	0	0	0	0	0.07
Arg	AGG	0	0	0.17	0.17	0.04
Arg	CGA	0	0	0	0	0.07
Arg	CGC	0.38	0.44	0.35	0.39	0.36
Arg	CGG	0	0	0	0	0.11
Arg	CGT	0.63	0.56	0.48	0.45	0.36
Asn	AAC	0.55	0.64	0.47	0.49	0.51
Asn	AAT	0.45	0.36	0.53	0.51	0.49
Cys	TGC	0.49	0.58	0.49	0.56	0.54
Cys	TGT	0.51	0.42	0.51	0.44	0.46
Glu	GAA	0.84	0.76	0.62	0.60	0.68
Glu	GAG	0.16	0.24	0.38	0.40	0.32
His	CAC	0.26	0.48	0.33	0.35	0.43
His	CAT	0.74	0.52	0.67	0.65	0.57
Ile	ATA	0	0	0	0	0.11
Ile	ATC	0.49	0.32	0.57	0.54	0.39
Ile	ATT	0.51	0.68	0.43	0.46	0.49
Phe	TTC	0.28	0.44	0.14	0.16	0.42
Phe	TTT	0.72	0.56	0.86	0.84	0.58
Ser	AGC	0.51	0.40	0.67	0.65	0.25
Ser	AGT	0.09	0.16	0.11	0.13	0.16
Ser	TCA	0.05	0.05	0.11	0.11	0.14
Ser	TCC	0.11	0.16	0.07	0.08	0.17
Ser	TCG	0.13	0.15	0.02	0.02	0.14
Ser	TCT	0.11	0.07	0.02	0.01	0.15

### Levels of expression of venom peptides are affected by the fusion tag

**Fig. 3:**
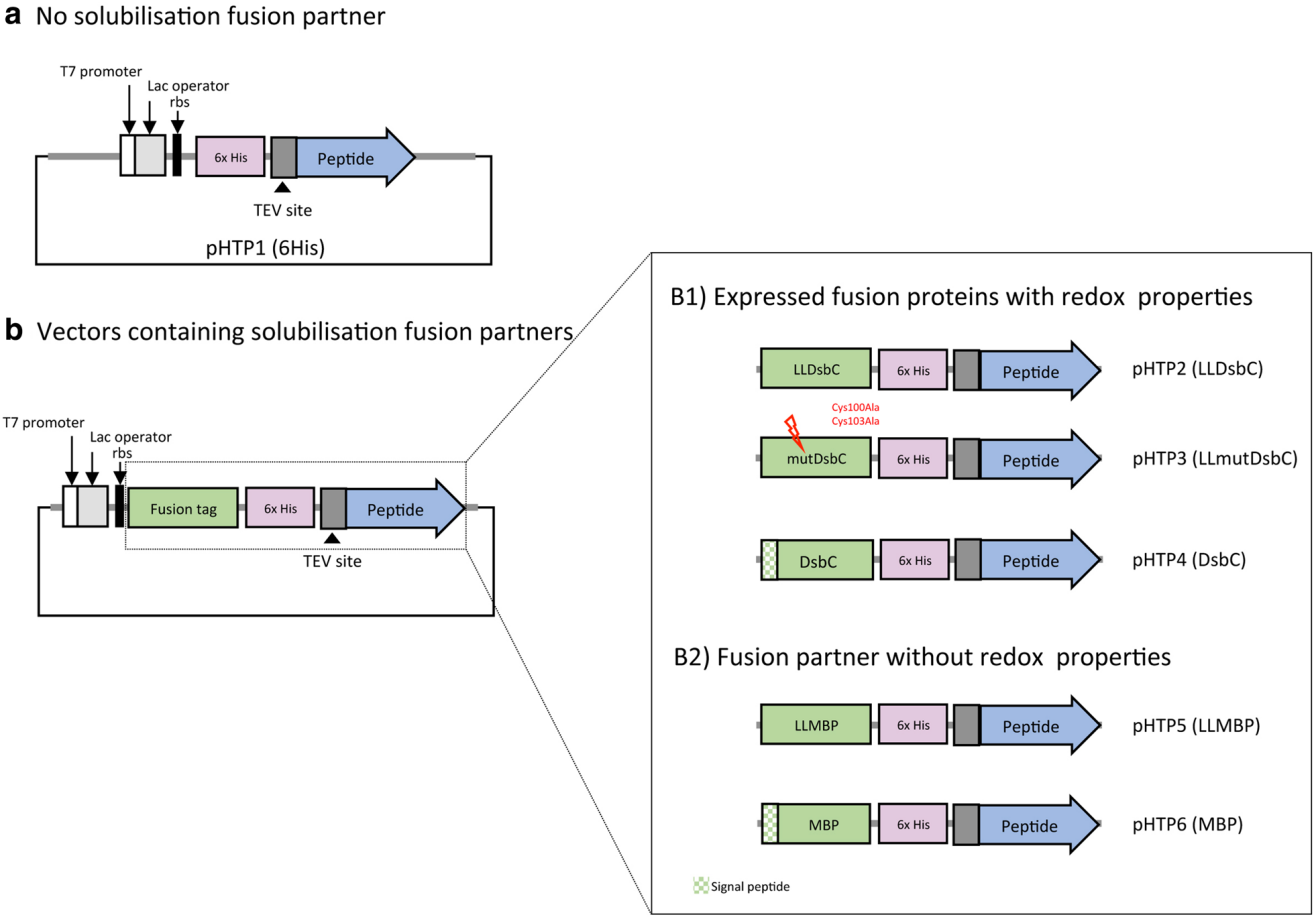
Schematic representation of the expression vectors that contain fusion tags with and without redox properties, which were used for cytoplasmic and periplasmic expression of venom peptides in *Escherichia coli*. All vectors include a T7 promoter, a ribosome binding site (rbs), a lac operator, a 6HIS tag for nickel affinity purification and a Tobacco Etch Virus (TEV) protease cleavage site. The 6HIS tag is N-terminal for pHTP1 vector (**a**) and internal for expression vectors including fusion tags (**b**). pHTP4 (DsbC) and pHTP6 (MBP) carry fusion tags containing a signal peptide (represented in crossed* green lines*) to target exportation of the fusion protein to the periplasm of *E. coli* cells. The inactive DsbC fusion partner, which contains two mutations at the catalytic site (C100A and C103A), was inserted into pHTP3 (LLmutDsbC)

Five novel vectors for recombinant protein expression in *E. coli* were constructed by inserting different fusion tags into the pHTP1 backbone. All fusion tags are to be inserted at the N-terminus of the recombinant peptides (Fig. 3). Two of the vectors encode fusion partners that contain a signal peptide to target venom peptide expression into the periplasm (pHTP4, pHTP6). The remaining fusion tags will lead to cytoplasmic recombinant protein expression (Fig. 3). In all cases a 6HIS tag was introduced to enable the downstream purification of the fusion proteins using immobilized-nickel affinity chromatography. A TEV (tobacco etch virus) protease cleavage site (ENLYFQ/G) was introduced in all synthetic genes to enable removal of the fusion partner. In addition to the 6HIS affinity tag alone (pHTP1) the 5 novel vectors include the disulphide isomerase DsbC or maltose binding protein (MBP). An inactive mutant derivative of DsbC was produced to try to discriminate the roles of DsbC in passive solubilisation (relating to fusion protein yield) and redox activity (relating to the yield of correctly folded target peptide). The schematic representation of all vectors use in this study is presented in Fig. 3.

**Fig. 4:**
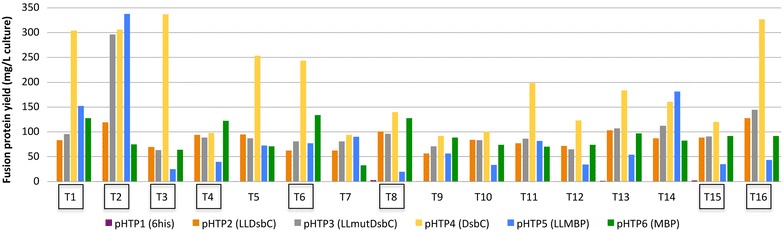
Yields of 96 purified recombinant fusion proteins originating from 16 different animal venom peptides in 6 fusions. Peptides are organized by increasing mass. Each fusion is represented by a *color* code. Yield is expressed in milligram of fusion per litre of culture. Fusion proteins were purified through IMAC and evaluated using the Labchip GXII (Caliper, USA). Peptides depicted in *boxes* were selected for the TEV cleavage experiment (see Fig. 5)

Sixteen well characterized venom peptides (including 7 that were part of the codon usage experiment described above) with different origins and representing different folds and cysteine bond patterns were selected for this study (see Table S3). The 16 synthetic genes were inserted into the six different expression vectors (see Table S2 and Fig. 3) generating a total of 96 recombinant plasmids. The 96 constructs were transformed in BL21 (DE3) pLysS. Recombinant *E. coli* strains were grown in auto-induction media to obtain high cell densities. After nickel affinity purification, systematic analysis of the Labchip GXII electropherograms was performed to determine the concentration of the purified proteins and compare the apparent molecular weight of the purified fusion proteins with their expected theoretical molecular weight. Data, presented in Fig. 4, revealed that the 16 peptides can indeed be produced using a fusion tag. Depending on the peptide and the fusion used, the levels of purified fusion proteins varied from zero (mostly when peptides were cloned in pHTP1) to more than 300 mg of purified fusion protein per litre of culture. Overall, smaller peptides seemed to be easier to produce than larger ones but there are several counter-examples (like T16 which is the largest peptide of the study). For peptides 2, 4, 7, 8, 9, 14 and 15, different vectors seemed to be appropriate for fusion protein expression but in most cases (13 out of 16) vector pHTP4 (DsbC) outperformed all other vectors. In contrast, without exception, soluble expression with 6HIS tag alone was always very low. The presence of the signal peptide lead to higher levels of expression for DsbC in all cases (pHTP4 versus pHTP2) doubling on average the amount of expressed fusion protein. For MBP (pHTP6 versus pHTP5) its presence gives a similar trend (10/16). For the other six peptides, either the cytoplasmic level was similar (1, 5, 11) or even significantly better (2, 7, 14). Overall out of 16 peptides, when the periplasmic DsbC was not the best option, it was the cytoplasmic (for peptides 2 and 14) or periplasmic (peptide 4) MBP that were the best options. Finally, the inactivation of DsbC biological function had no effect in expression levels of venom peptide fusion proteins, as expression of pHTP2 and pHTP3 were, in general, very similar.

### Fusion cleavage, peptide yield and correct oxidation state is mainly affected by the fusion partner and DTT concentration in TEV cleavage buffer

Optimal TEV cleavage conditions to release target peptides from fusion tags are affected by several parameters including enzyme/substrate ratio, buffer composition, incubation period and temperature. To investigate which conditions would lead to the best yield of folded venom peptides, eight peptides were selected from the list of 16 produced in the previous experiment (See Additional file 3: Table S3, peptides in italic, and Fig. 4, peptides in boxes). Because the DsbC fusion partner outperformed other tags in terms of fusion protein yields and general applicability, these constructs were selected for the TEV cleavage optimisation study. The only parameter that turned out to be critical and therefore was was fine-tuned in this experiment was the DTT concentration (0, 0.1, 0.5 and 2 mM DTT) present in the cleavage buffer. Indeed, while the TEV protease requires reducing conditions for optimum cleavage, an excessive concentration of DTT could lead to the reduction of the peptides’ intra-disulphide bridges and loss of folding and biological activity.

**Fig. 5:**
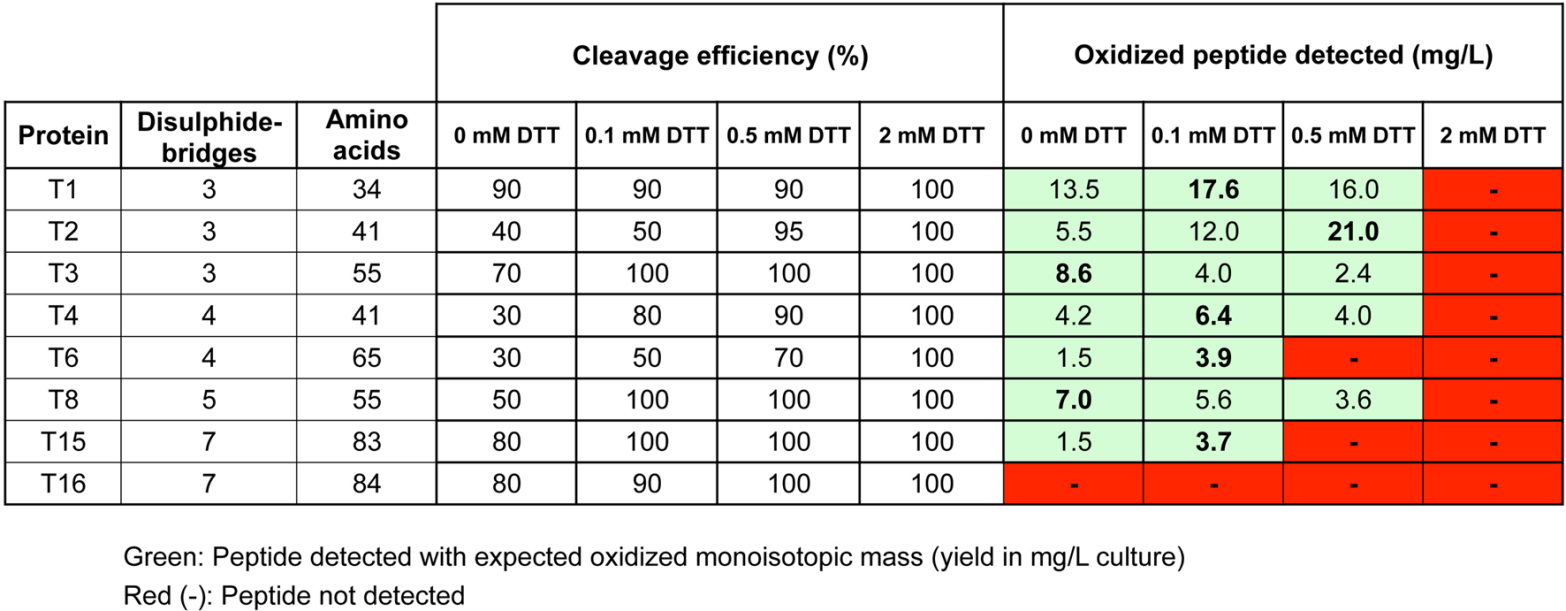
TEV cleavage efficacy in various concentrations of DTT. The cleavage efficiency represents the percentage of fusion cleaved for each DTT concentration (0–2 mM) quantified by Labchip GXII (Caliper, USA) and depicted in percentages. The correct oxidation state of the purified peptide was confirmed by LC–MS (*green* mass corresponds to oxidized peptide, *red* no peptide detected). When a correct mass is detected the yield of peptide per litre of culture quantified by integration of the LC peaks is indicated in the well

After an 18 h incubation period, an aliquot of the TEV-fusion peptide mixture was acidified. A fraction (“before” versus “after” TEV cleavage) was loaded on the Caliper to determine the cleavage efficiency and a sample was analysed on the LC–MS to quantify the toxin and confirm its correct oxidation state. Cleavage efficiency, mass analysis and peptide yields are summarized in Fig. 5. Cleavage occurred in the 32 conditions tested in the assay (ranging from 30 to 100% efficiency). As expected, cleavage was not complete in the absence of DTT and complete with the highest concentration of DTT. Out of eight peptides, seven could be detected oxidized in mg quantities per litre of culture. From the 32 samples, 19 gave the correct oxidized mass on the LC–MS with various yields depending on the DTT concentration during cleavage. For the 13 samples left, no peaks were detected on the LC–MS. From the seven peptides correctly detected in various DTT concentration, four gave the highest recovery in 0.1 mM DTT, while two needed no DTT and one needed 0.5 mM DTT for optimum recovery (Fig. 5, in bold). A DTT concentration of 0.1 mM appeared to be the best compromise to be kept for the following experiments and the production pipeline of the VENOMICS Project. Aliquots of the 7 peptides were concentrated to 2 and 4 mg/mL and subjected to the same experiment with 0.1 mM DTT to confirm that cleavage would be possible in these conditions. On average, the efficiency dropped by 20% but occurred in all cases (data not shown).

**Fig. 6:**
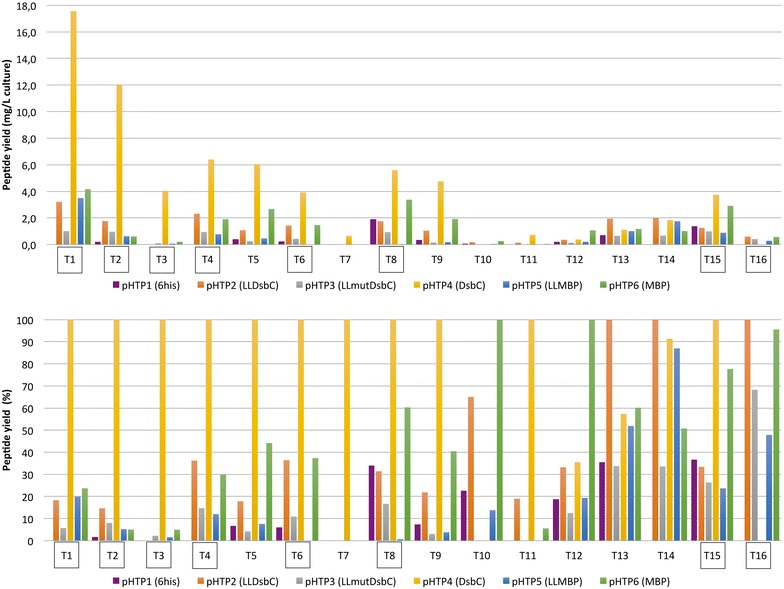
Yields of 96 purified recombinant peptides after tag removal. Peptides are organized by increasing mass. Each original fusion tag used to express each peptide is represented by a *color code* (identical to Fig. 4). Yield is in milligram of oxidized peptide per litre of culture. The correct oxidation state of the purified peptide was confirmed by LC–MS and the yield of peptide per litre of culture quantified by integration of the LC peaks. **a** Concentration in mg/L of culture. **b** Yield of peptide is presented in percentage relative to the best condition to better visualize the low expressing peptides. Peptides depicted in *boxes* were selected for the TEV cleavage experiment (Fig. 5)

Following the optimization of cleavage conditions, the 96 purified fusion proteins (16 in 6 vectors, see Fig. 4) were cleaved in the presence of 0.1 mM DTT at the concentration of the purified pools (ranging mostly from 0.2 to 2 mg/mL) following the protocol described above. After cleavage and acidification an aliquot of the 96 samples was analysed by LC–MS to confirm the correct molecular mass, yield and the oxidation state of the final recombinant venom peptide. When detected, the 96 recombinant peptides had molecular masses in agreement with the expected masses given fully oxidized cysteine residues (See accompanying article for more details on the mass Spectrometry analysis); the reduced forms of the proteins were never detected (data not shown), probably due to precipitation of incorrectly oxidized peptides during cleavage and acidification steps. The final yield for the 96 constructs, presented in Fig. 6, was expressed as absolute peptide final yield in mg/L culture or normalized to 100% for each peptide relative to the vector used for expression. The data (Fig. 6) revealed that all 16 peptides under study could be produced recombinantly, but at different levels. Similar to the yield obtained for the fusion variants (Fig. 4), a general trend suggests that the shorter peptides are easier to produce than the longer ones. The final venom peptide yield varied greatly with a 50-fold difference between the worst case (0.3 mg/L, T10 in pHTP6) and the best case (17.6 mg/L, T1 in pHTP4). The dataset also revealed that there was a significant drop in yields following fusion tag cleavage. This is probably due to the harsh recovery condition after cleavage (acidification in 5% acetonitrile, 0.1% formic acid) where on top of the TEV protease, any cleaved misfolded peptide precipitates. As expected from the fusion yields, even if recovery is high, peptides produced with pHTP1 gave the lowest quantities of peptides overall (0.4 mg/L on average, with a maximum of 1.9 mg/L for T8). In contrast, peptides produced with pHTP4 (DsbC) reached the best final peptide yields for 11 of 16 peptides, and of these (with the exception of T11) yields were more than 2 mg/L for each peptide (4.6 mg/L average). Overall, DsbC fusions (for either periplasmic or cytoplasmic expression) successfully produced 14 out of 16 venom peptides. Furthermore, one peptide (T7) could only be produced in the periplasm, using the DsbC fusion partner, from the pHTP4 vector. For peptides produced preferentially from other vectors (T10, 12, 13, 14, 16), yields do not surpass 2 mg/L, highlighting the robust expression from the pHTP4 vector. For these five peptides, highest yields were achieved with cytoplasmic expression and a DsbC fusion partner in three cases, followed by periplasmic expression with the MBP fusion partner in two cases. In most cases (except T13,T14 and T16), the fusion exported to the periplasm (for either DsbC or MBP) outperformed its cytoplasmic equivalent, indicating that at least part of the folding could occur in the periplasm and part could occur ex vivo during the purification. A striking demonstration that DsbC (and probably MBP) acts, in part, as a passive solubilising agent inside the bacteria is the fact that while the fusion yields between the DsbC and the mutated DsbC constructs were very similar for most peptides (Fig. 4), after cleavage and recovery, the overall yield of active peptide is on average three times higher in the case of the redox-active DsbC fusion than with its mutated DsbC counterpart. The detail of the quantitative values has been summarized in Additional file 7: Table S7.

### The nature of the C-terminal (P1′) residue of the TEV cleavage site does not significantly affect cleavage efficacy

**Fig. 7:**
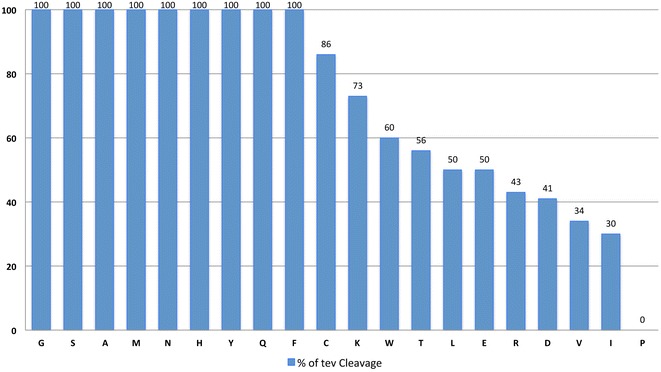
TEV protease cleavage efficiency of Kin17 with 20 different amino acids located at position P1′. The amino acids are organized from the easiest to the most difficult ones to cleave. Values are in percentages

The N-terminus of some venom peptides can contribute to their receptor binding sites. Thus, it is possible that the introduction of a single extra residue at the N-terminus of the peptide may affect its biological activity [15]. The canonical TEV protease recognition site requires a Gly or Ser residue at its C-terminus (P1′ position), leaving a non-native Ser or Gly residue at the N-terminus of the target peptide after tag removal. A previous study [30] suggested that, with the exception of proline, all the amino acid side-chains could be accommodated in the P1′ position of a TEV protease recognition site with little impact on the efficiency of processing. The analysis was, however, performed in optimal TEV buffer conditions. In order to be time-efficient, the venom peptide production pipeline can not accommodate additional steps such as buffer exchange into optimal TEV conditions. Thus, the capacity of TEV protease to act in the IMAC elution buffer (Tris 50 mM, NaCl 300 mM, Imidazole 250 mM, DTT 0.1 mM, pH8) a non-optimum buffer for TEV proteolysis, was investigated. The TEV_SH_ protease used in this study [29] was selected because it is easy to overproduce and purify in *E. coli* at very high quantities (up to 100 mg/L culture). However, the cleavage specificity of this recombinant derivative of TEV protease remains unknown, in particular when various amino acids occupy the P1′ position of its recognition site (Dr H. Berglund, personal communication). Thus, to explore the activity of this TEV_SH_ protease in non-optimal conditions and when the P1′ position of the protease recognition site is varied, 20 test-cleavage fusion protein sequences were produced. Each fusion protein contained an N-terminal 6HIS tag, an internal TEV recognition sequence, each containing a different amino acid at the P1´ position, fused C-terminally to a truncated form of the DNA/RNA-binding protein Kin17 from *Homo sapiens*. Proteins were purified and subjected to TEV protease cleavage in the same conditions as those used to cleave the 96 fusion tags (see above). The data, presented in Fig. 7, confirm previous data collected [30] and suggest that, with the exception of proline (the probability of having a proline in position 1 of naturally-sourced venom peptides is low), all other residues can be accommodated at the P1′ position of the TEV protease recognition site while retaining some cleavage activity. However, consideration should be given to peptides with an N-terminal Trp, Thr, Leu, Glu, Arg, Asp, Val or Ile, where cleavage efficiency dips to 60% or less. In these cases a compromise between cleavage efficiency/production yield may need to be reached, depending on how well the peptide expresses.

Note that in contrast to venom peptides, Kin17 does not contain cysteine residues. Thus, the successful cleavage yield when a cysteine residues is in position 1 (86%) obtained with Kin17 needs to be confirmed for the cases of peptides with a cysteine in position 1 that would probably be involved in a disulphide bridge in the native protein.

## Discussion

Today de novo gene synthesis is replacing the classic cloning approaches for the construction of transgenes and thus it is critical to develop effective gene design algorithms that could sustain high levels of heterologous gene expression [31]. Effective design methods may require attending to particular properties of different protein families. Thus, the intrinsic abundance of cysteine residues in venom peptides is potentially a critical factor that may affect the recombinant expression of such peptides. Here, by designing and synthesizing 72 individual genes encoding 24 different peptides, nucleic acid sequence differences affecting the levels of soluble expression of venom peptides were identified. Highly expressing gene variants produced up to twice the levels of recombinant protein when compared with low expressing ones. Factors affecting expression levels were identified by comparing the codon usage of high and low expressing variants. The data revealed that most of the variation in expression can be explained, primarily, by differences in the frequency of cysteine codons but also, at a lower level, for arginine and isoleucine. Thus, data presented here reveal that high levels of expression of venom peptides require a similar usage of the two cysteine codons Cys-TGT and Cys-TGC. It is now well established that high translation rates contribute to deplete the cellular translational machinery [32]. Considering the levels of expression of the heterologous proteins reported in this study we estimate that between 25 and 40% of the total proteins produced by the bacterial cell comprise recombinant fusion polypeptides. Overexpression of recombinant genes in *E. coli* leads to a significant change in the amino acids being used for protein synthesis in favour of the recombinant protein. In the particular case of venom peptides, cysteine is a highly frequent residue being around four times more frequent in the recombinant fusion genes than in regular *E. coli* genes. Thus recombinant *E. coli* strains expressing venom peptides at high levels will require a similar usage of both cysteine codons most likely to avoid depletion of one relative to the other. Thus if one codon was present in higher frequencies then this will be more easily depleted within the cell and will become the limiting codon for rate of gene synthesis. Thus, the data suggests that codon usage is indeed one of the key determinants of expression yield. Regardless of the mechanism by which codon bias affects expression, systematic analysis of the relationship between gene sequences and expression will be a powerful tool to refine our design algorithms, both for *E. coli* and other expression hosts.

Data presented here revealed that the best way to express high yields of folded active animal venom peptides is their expression in the periplasm of *E. coli*. One excellent example of the production potential using periplasmic expression is the snake venom peptide, MT7 (peptide 6 in this study), containing 4 disulphide bonds. MT7 was previously expressed using DsbC as a fusion partner in the cytoplasm of *E. coli* [11]. While cytoplasmic expression achieved high yields of the DsbC fusion protein, after cleavage the peptide could only be purified to a yield of 0.007 mg/L culture. Here, by exporting the fusion protein to the periplasm, the yield of soluble, oxidized MT7 was increased 500-fold (to 3.9 mg/L culture). Note that for the cytoplasmic expression of MT7 with a DsbC fusion (the equivalent of the constructs of MT7 used in the previous study), most parameters have been modified between both studies; i.e. codon usage, vector backbone, culture and cleavage conditions… These modifications alone increased dramatically the yield of MT7 compared to our previous study stressing out that every steps of the optimized protocol detailed in this study can have a drastic impact on the final yield of oxidized peptides. In this study, DsbC was found to be a much more efficient fusion partner to express venom peptides in the periplasm of *E. coli* than MBP. The encouraging results obtained with DsbC may derive from its excellent solubilisation potential but, more importantly, from its isomerase and chaperonin activities (which MBP lacks) that may promote the folding of venom peptides. In the cytoplasmic compartment, the redox-inactive tags used (an inactive mutant derivative of DsbC) lead to the production of similar yields of recombinant peptides as the wild type DsbC. This suggests that redox properties of the fusion tag do not affect solubility and folding of animal venom peptides during the expression in the cytoplasm of *E. coli* and therefore confirm that oxidation occurs, primarily, ex vivo [8]. During the production and purification of the proteins, DsbC is improving the solubility of venom peptides rather than assisting the peptides to reach their native oxidized form. Nevertheless, during cleavage and in the presence of DTT, DsbC probably also acts as an isomerase as the yield of peptide varies greatly depending on the DTT concentration. Additionally, peptide yields from the inactive mutant derivative of DsbC are much lower than the two other redox-active DsbC constructs. By using the DsbC fusion partner we were able to generate high yields of folded, putatively biologically active, venom peptides in the periplasm of *E. coli*. Mass spectrometry (coupled with liquid chromatography) not only confirmed the exact oxidized mass of all species, but also the correct connections of the disulphide bridges within the 16 toxins of this study. In the case of MT7, where an activity test was available, the activity of the purified peptide was confirmed. Data presented here, using TEV variant protein TEV_SH_, a highly soluble TEV mutant, confirmed that all residues (except proline) could be accommodated in the P1′ position with little or no impact on the efficiency of processing, even in a non-optimized cleavage condition. Therefore, the removal of the associated fusion tag with TEV protease (using the shortened recognition site: ENLYFQ only) could effectively produce a venom peptide with exactly the same sequence properties and biological activity as that of non-recombinant molecules.

While the scope of this study was to identify the best unique protocol to produce with a high-throughput pipeline correctly oxidized toxins in *E. coli*, we believe that this work could be of use for a wider community. The results shed light, for scientists working on a more limited number of toxins, on the parameters that could be explored in the case that our best protocol wouldn’t be successful on their targets. As demonstrated in the article, the main factors affecting the results are the choice of codon usage (if the genes have to be synthetized), the choice of fusion tags (a good alternative to periplasmic DsbC could be periplasmic MBP or the cytoplasmic DsbC or MBP) and finally, sometimes really crucial, the concentration of DTT in the TEV cleavage buffer.

## Conclusions

There is an urgent need to develop effective methods to express large libraries of recombinant disulphide-rich peptides, whether sourced from animal venoms or artificial banks, which could be applied in innovative screening platforms for the discovery of novel therapeutics. *E. coli* is a highly robust heterologous host but it displays substantial limitations for the production of eukaryotic proteins with multiple disulphide bridges, furthermore when thousands of peptides are in the scope of the project. Here we have analysed how to modulate the levels of expression, solubility and oxidation of animal venom peptides produced in bacteria. This report shows that under optimized conditions *E. coli* is a pertinent host for the expression of biologically active animal venom peptides even using a single protocol to produce peptides with variable length, fold and cysteine pattern. Genes encoding venom peptides that are expressed at higher levels in *E. coli* present a codon usage that suggest a similar representation of the two Cys codons. This study demonstrates that the expression of venom peptides in the bacterial periplasm with the help of a DsbC fusion is one of the best options to purify milligram yields of active peptides, although the data suggest that peptide folding by DsbC occurs mainly ex vivo. Finally, with the exception of Pro, TEV protease can effectively tolerate any of the N-terminal amino acids located in venom peptides, suggesting that retention of the native peptide N-terminus is compatible with an effective protease cleavage.

The findings reported here have been applied to the construction of a high-throughput platform for the expression of venom peptides in *E. coli*, and the production of the largest bank of animal venom peptides known, under the VENOMICS Project, and is reported in the accompanying paper.

## Additional files

**Additional file 1: Table S1.** Properties of venom peptides selected for this study and yield after recombinant expression of three gene variants per peptide as DsbC fusions.

**Additional file 2: Table S2.** Properties of the novel prokaryotic expression vectors.

**Additional file 3: Table S3.** Properties of venom peptides selected for the study of the impact of protein fusions on soluble yield.

**Additional file 4: Table S4.** Sequence characteristics of human Kin17 (KN17) protein and primer sequences used to produce 20 protein variants with different N-terminal amino acids.

**Additional file 5: Table S5.** Codon frequency used to design the variants A, B and C of 24 optimized genes encoding venom peptides.

**Additional file 6: Table S6.** Codon usage of genes encoding high and low expressing variants encoding either venom peptides or their respective fusion proteins.

**Additional file 7: Table S7.** Properties of venom peptides selected for this study, fusion and final toxin yields after recombinant expression and purification.

## Abbreviations

ACN: acetonitrile
CAI: codon adaptation index
DTT: dithiothreitol
Dsb (A or C): disulfide oxidoreductase (A or C)
FP7: Framework Program 7 (European funding)
HTP: high-throughput
L. C: liquid chromatography
TEV: tobacco etch virus

## References

[1] Escoubas P, King GF (2009). Venomics as a drug discovery platform. Expert Rev Proteom..

[2] Lewis RJ, Garcia ML (2003). Therapeutic potential of venom peptides. Nat Rev Drug Discov.

[3] Fernandes-Pedrosa MF, Félix-Silva J, Menezes Y (2013). Toxins from venomous animals: gene cloning, protein expression and biotechnological applications. Integr View Mol Recognit Toxinologu Anal Proced Biomed Appl..

[4] Welch M, Villalobos A, Gustafsson C, Minshull J (2011). Designing genes for successful protein expression. Methods Enzymol.

[5] Henaut A, Danchin A, Neidhart FC, Curtiss RI, Ingraham J, Lin E, Brooks Low K (1996). Analysis and predictions from *Escherichia coli* sequences. *Escherichia coli* Salmonella typhimurium: cellular and molecular biology.

[6] Fuglsang A (2003). The effective number of codons for individual amino acids: some codons are more optimal than others. Gene.

[7] Welch M, Villalobos A, Gustafsson C, Minshull J (2009). You’re one in a googol: optimizing genes for protein expression. J R Soc Interface.

[8] Saez NJ, Nozach H, Blemont M, Vincentelli R. High throughput quantitative expression screening and purification applied to recombinant disulfide-rich venom proteins produced in *E. coli*. J Vis Exp. 2014;(89):e51464. 10.3791/51464 PMC4692350 25146501

[9] Saez NJ, Vincentelli R (2014). High-throughput expression screening and purification of recombinant proteins in *E. coli*. Methods Mol Biol.

[10] Clement H, Flores V, Diego-Garcia E, Corrales-Garcia L, Villegas E, Corzo G (2015). A comparison between the recombinant expression and chemical synthesis of a short cysteine-rich insecticidal spider peptide. J Venom Anim Toxins Incl Trop Dis..

[11] Nozach H, Fruchart-Gaillard C, Fenaille F, Beau F, Ramos OHP, Douzi B (2013). High throughput screening identifies disulfide isomerase DsbC as a very efficient partner for recombinant expression of small disulfide-rich proteins in *E. coli*. Microb Cell Fact.

[12] Costa SJ, Almeida A, Castro A, Domingues L (2014). Fusion tags for protein solubility, purification and immunogenicity in *Escherichia coli*: the novel Fh8 system. Front Microbiol..

[13] Parks TD, Leuther KK, Howard ED, Johnston SA, Dougherty WG (1994). Release of proteins and peptides from fusion proteins using a recombinant plant virus proteinase. Anal Biochem.

[14] Dougherty WG, Carrington JC, Cary SM, Parks TD (1988). Biochemical and mutational analysis of a plant virus polyprotein cleavage site. EMBO J.

[15] Karbat I, Turkov M, Cohen L, Kahn R, Gordon D, Gurevitz M (2007). X-ray structure and mutagenesis of the scorpion depressant toxin LqhIT2 reveals key determinants crucial for activity and anti-insect selectivity. J Mol Biol.

[16] Wu G, Wolf JB, Ibrahim AF, Vadasz S, Gunasinghe M, Freeland SJ (2006). Simplified gene synthesis: a one-step approach to PCR-based gene construction. J Biotechnol.

[17] Xiong A-S, Peng R-H, Zhuang J, Gao F, Li Y, Cheng Z-M (2008). Chemical gene synthesis: strategies, softwares, error corrections, and applications. FEMS Microbiol Rev.

[18] Hartley JL, Temple GF, Brasch MA (2000). DNA cloning using in vitro site-specific recombination. Genome Res.

[19] Vincentelli R, Cimino A, Geerlof A, Kubo A, Satou Y, Cambillau C (2011). High-throughput protein expression screening and purification in *Escherichia coli*. Methods.

[20] Studier FW (2005). Protein production by auto-induction in high density shaking cultures. Protein Expr Purif.

[21] Klint JK, Senff S, Saez NJ, Seshadri R, Lau HY, Bende NS (2013). Production of recombinant disulfide-rich venom peptides for structural and functional analysis via expression in the periplasm of *E. coli*. PLoS ONE.

[22] Cardoso FC, Dekan Z, Rosengren KJ, Erickson A, Vetter I, Deuis JR (2015). Identification and characterization of ProTx-III [μ-TRTX-Tp1a], a new voltage-gated sodium channel inhibitor from venom of the tarantula thrixopelma pruriens. Mol Pharmacol.

[23] Anangi R, Rash LD, Mobli M, King GF (2012). Functional expression in *Escherichia coli* of the disulfide-rich sea anemone peptide APETx2, a potent blocker of acid-sensing ion channel 3. Mar Drugs..

[24] Bende NS, Dziemborowicz S, Mobli M, Herzig V, Gilchrist J, Wagner J (2014). A distinct sodium channel voltage-sensor locus determines insect selectivity of the spider toxin Dc1a. Nat Commun..

[25] Bende NS, Dziemborowicz S, Herzig V, Ramanujam V, Brown GW, Bosmans F (2015). The insecticidal spider toxin SFI1 is a knottin peptide that blocks the pore of insect voltage-gated sodium channels via a large b-hairpin loop. FEBS J.

[26] Meng E, Cai TF, Li WY, Zhang H, Liu YB, Peng K (2011). Functional expression of spider neurotoxic peptide Huwentoxin-I in *E. coli*. PLoS ONE.

[27] Saez NJ, Mobli M, Bieri M, Chassagnon IR, Malde AK, Gamsjaeger R (2011). A dynamic pharmacophore drives the interaction between Psalmotoxin-1 and the putative drug target acid-sensing ion channel 1a. Mol Pharmacol.

[28] Yang S, Xiao Y, Kang D, Liu J, Li Y, Undheim E (2013). Discovery of a selective NaV1.7 inhibitor from centipede venom with analgesic efficacy exceeding morphine in rodent pain models. Proc Natl Acad Sci USA.

[29] Van Den Berg S, Löfdahl PÅ, Härd T, Berglund H (2006). Improved solubility of TEV protease by directed evolution. J Biotechnol.

[30] Kapust RB, Tözsér J, Copeland TD, Waugh DS (2002). The P1′ specificity of tobacco etch virus protease. Biochem Biophys Res Commun..

[31] Czar MJ, Anderson JC, Bader JS, Peccoud J (2009). Gene synthesis demystified. Trends Biotechnol.

[32] Dong H, Nilsson L, Kurland CG (1995). Gratuitous overexpression of genes in *Escherichia coli* leads to growth inhibition and ribosome destruction. J Bacteriol.

